# Supplementary material for: Light regulates xylem cell differentiation via PIF in Arabidopsis
Source: Cell Rep. 2022 Jul 19;40(3):111075. doi: 10.1016/j.celrep.2022.111075 (PMC9638722; doi:10.1016/j.celrep.2022.111075)
Supplement: Document S1. Figures S1 and S2 [file mmc1.pdf]

Cell Reports, Volume *40*

## Supplemental information

# Light regulates xylem cell differentiation

# via PIF in *Arabidopsis*

**Shraboni Ghosh, Joseph F. Nelson, Geoffrey M.C. Cobb, J. Peter Etchells, and Miguel de Lucas**

# Light regulates xylem cell differentiation via PIF in Arabidopsis

Shraboni Ghosh[a], Joseph F. Nelson[a], Geoffrey Cobb[ab], J. Peter Etchells[a] and Miguel de Lucas[a]

## SUPPLEMENTARY FIGURES

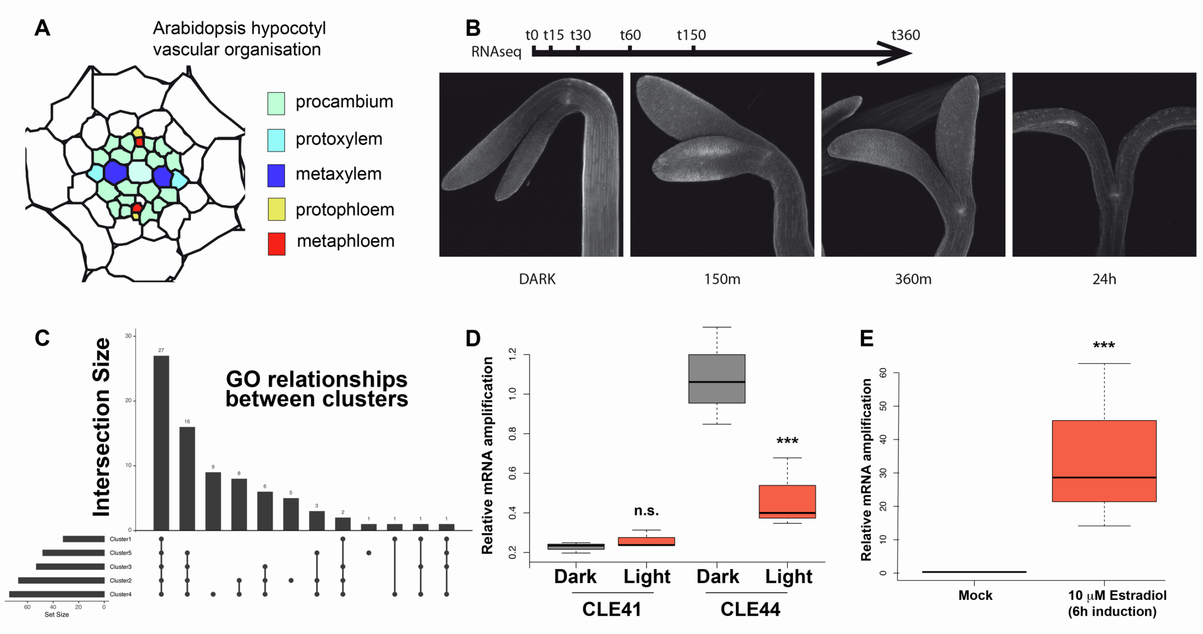


**Figure S1. Related to Figure 1, 2 and 4**

(A) Cartoon of a cross section of the Arabidopsis hypocotyl showing the different cell types forming the vascular tissue.

(B) Diagrammatic representation of the RNAseq experimental set up. Samples were taken at different time points over the course of 5-day old seedling deetiolation (Dark, 15, 30, 60, 150 and 360min, three replicates per time point). Related to Figure 1J and Data S1.

(C) Upset plot describing the relationships between enriched gene-ontology categories across the five different gene clusters. Related to Data S2.

(D) Comparison between *CLE41 and CLE44* expression levels in seedlings grown in the dark and after 6h of light. Data are representative of three independent experiments and three technical replicates per pair of primers. Values represent mean of expression ± SD. Letters indicate Student's t.test ($*p < 0.05$, $**p < 0.01$, $***p < 0.001$, ns = not significant). Related to Figure 1 and 2.

(E) Confirmation of *CLE44* induction in XVE:*CLE44* seedlings. Data are representative of three independent experiments and three technical replicates per pair of primer. Values represent mean of expression ± SD. Letters indicate Student's t.test ($*p < 0.05$, $**p < 0.01$, $***p < 0.001$, ns = not significant). Related to Figure 2 and 4.

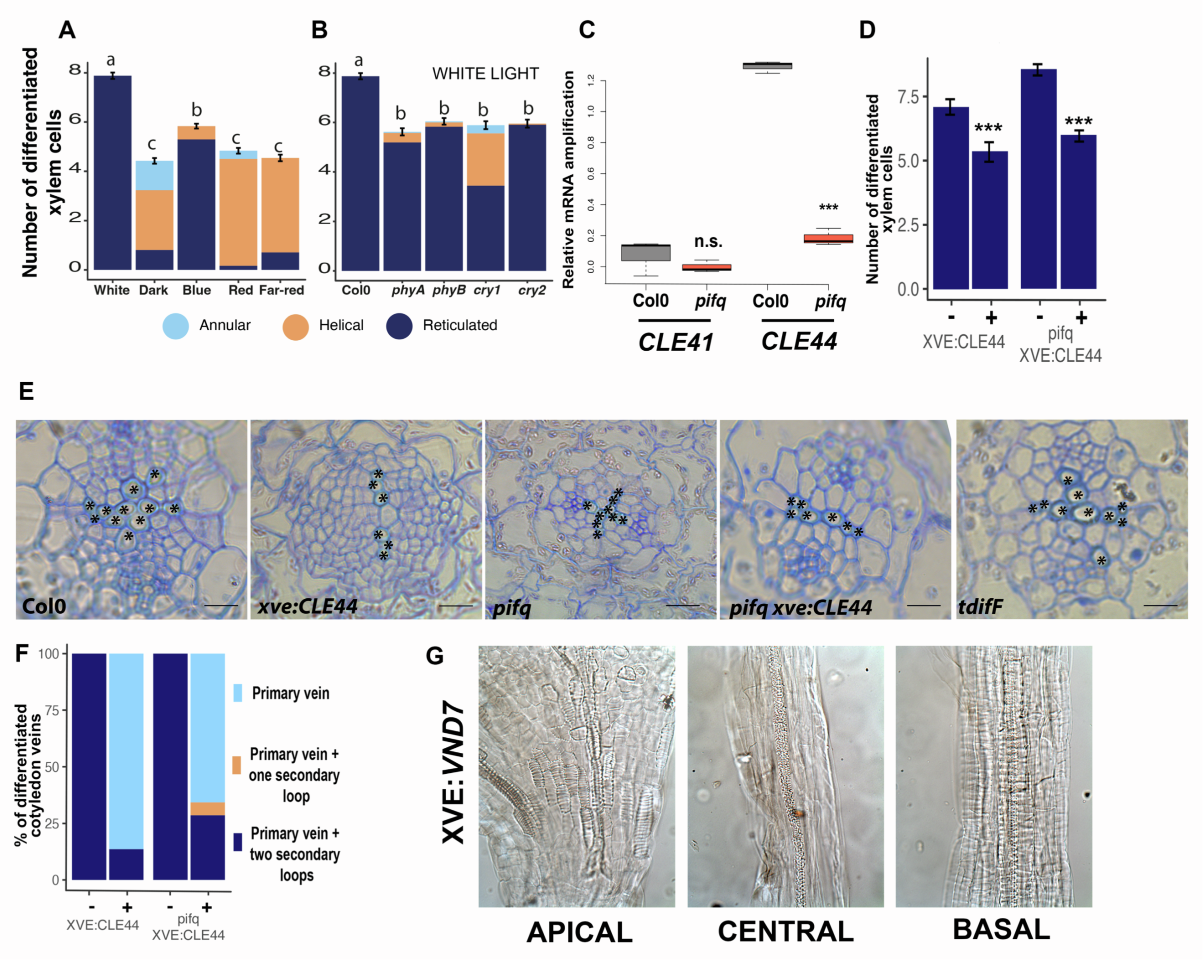


**Figure S2. Related to Figure 4.**

(A) Differences in hypocotyl xylem differentiation between seedlings treated with different wavelengths of monochromatic light. Values (n>24) represent mean of differentiated cells ± SE. Letters represent ANOVA + Tukey HSD statistical test. Data S5 contains details of the sample size, mean, SE values and ANOVA + Tukey HSD comparisons.

(B) Differences in hypocotyl xylem differentiation between photoreceptor mutants grown under white light. Values (n>18) represent mean of differentiated cells ± SE. Letters represent ANOVA + Tukey HSD statistical test. Data S5 contains details of the sample size, mean, SE values and ANOVA + Tukey HSD comparisons.

(C) Differences between *CLE41* and *CLE44* expression levels in Col0 (WT) and *pifq* mutant seedlings grown in the dark. Data are representative of three independent experiments and three technical replicates per pair of primer. Values represent mean of expression ± SD. Letters indicate Student's t.test (∗p < 0.05, ∗∗p < 0.01, ∗∗∗p < 0.001, ns = not significant).

(D) Differences in hypocotyl xylem differentiation between XVE:*CLE44* and XVE:*CLE44* in *pifq* seedlings grown in the light and in the presence of mock and 17ß-estradiol. Values (n>9) represent mean of differentiated

cells ± SE. Letters represent ANOVA + Tukey HSD statistical test. Data S5 contains details of the sample size, mean, SE values and ANOVA + Tukey HSD comparisons.

(E) Hypocotyl plastic cross sections showing the differences in xylem cell differentiation between Col0, XVE:*CLE44*, *pifq*, XVE:*CLE44 pifq* and *tdif-F* grown for 5-days in the presence of 17ß-estradiol. Xylem differentiated cells are indicated with and asterisk.

(F) Differences (as percentage) in cotyledon vein differentiation of photoreceptor mutants grown in the light. Data S5 contains details of the sample size and percentages

(G) DIC image showing the ectopic xylem differentiation caused by the induction of VND7 in different regions (apical, central and basal) of dark-grown hypocotyls.
