# Supplementary material for: Light regulates xylem cell differentiation via PIF in Arabidopsis
Source: Cell Rep. 2022 Jul 19;40(3):111075. doi: 10.1016/j.celrep.2022.111075 (PMC9638722; doi:10.1016/j.celrep.2022.111075)
Supplement: Document S2. Article plus supplemental information [file mmc7.pdf]

Cell Reports

Light regulates xylem cell differentiation via PIF in *Arabidopsis*

Graphical abstract

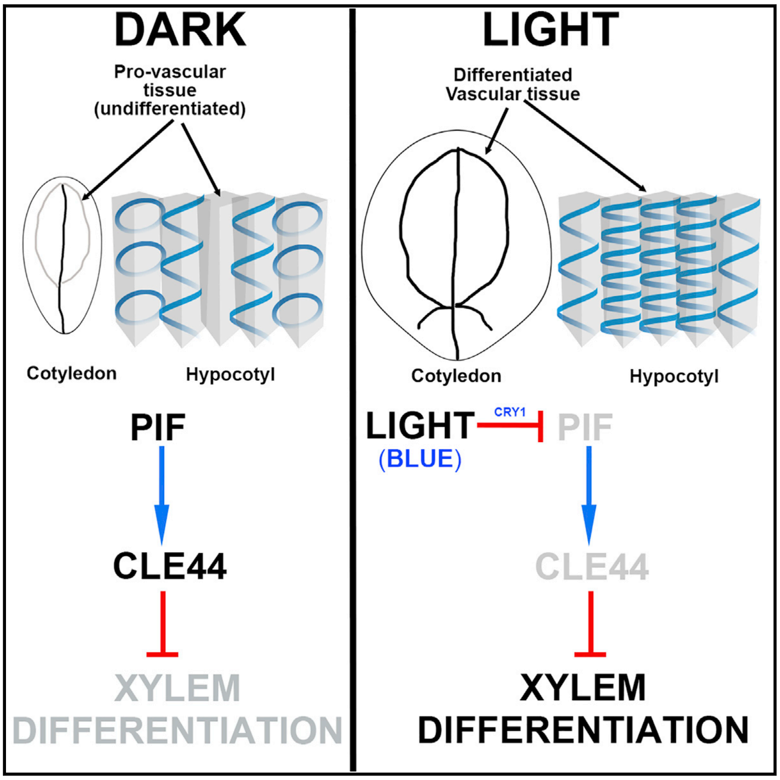

Highlights

- Active CLE peptide TDIF inhibits xylem differentiation in etiolated seedlings
- The expression of the TDIF precursor CLE44 is rapidly inhibited by light
- PIF transcription factors are necessary for TDIF expression in the dark
- Blue light signaling prevents TDIF expression, which promotes xylem differentiation

Authors

Shraboni Ghosh, Joseph F. Nelson, Geoffrey M.C. Cobb, J. Peter Etchells, Miguel de Lucas

Correspondence

miguel.de-lucas@durham.ac.uk

In brief

As water is essential for photosynthesis, plant vascular differentiation must be orchestrated with environmental fluctuations. Ghosh et al. report that xylem cell differentiation is regulated by light in a mechanism that involves the fine-tune regulation of the TDIF precursor *CLE44* by PIF transcription factors.

Ghosh et al., 2022, Cell Reports *40*, 111075
July 19, 2022 © 2022 The Author(s).
https://doi.org/10.1016/j.celrep.2022.111075

CellPress

Cell Reports

CellPress
OPEN ACCESS

Report

Light regulates xylem cell differentiation via PIF in *Arabidopsis*

Shraboni Ghosh,[1] Joseph F. Nelson,[1] Geoffrey M.C. Cobb,[1,2] J. Peter Etchells,[1] and Miguel de Lucas[1,3,*]
[1]Department of Biosciences, Durham University, Durham DH1 3LE, UK
[2]Present address: Center for Integrative Genomics, Faculty of Biology and Medicine, University of Lausanne, 1015 Lausanne, Switzerland
[3]Lead contact
*Correspondence: miguel.de-lucas@durham.ac.uk
https://doi.org/10.1016/j.celrep.2022.111075

SUMMARY

The balance between cell proliferation and differentiation in the cambium defines the formation of plant vascular tissues. As cambium cells proliferate, subsets of daughter cells differentiate into xylem or phloem. TDIF-PXY/TDR signaling is central to this process. TDIF, encoded by *CLE41* and *CLE44*, activates PXY/TDR receptors to maintain proliferative cambium. Light and water are necessary for photosynthesis; thus, vascular differentiation must occur upon light perception to facilitate the transport of water and minerals to the photosynthetic tissues. However, the molecular mechanism controlling vascular differentiation in response to light remains elusive. In this study we show that the accumulation of PIF transcription factors in the dark promotes TDIF signaling and inhibits vascular cell differentiation. On the contrary, PIF inactivation by light leads to a decay in TDIF activity, which induces vascular cell differentiation. Our study connects light to vascular differentiation and highlights the importance of this crosstalk to fine-tune water transport.

INTRODUCTION

Water transport is required for photosynthesis; thus, plant vascular development must proceed such that water demands can be met during formation of new organs. Xylem and phloem, the vascular tissues, are defined in the cambium via precise regulation of the balance between cell proliferation and differentiation. TDIF-PXY/TDR signaling is central to this process (Etchells et al., 2016). Peptide ligand TDIF, encoded by *CLE41* and *CLE44* (Ito et al., 2006), activates PXY/TDR receptors to maintain proliferation. Cambium cells differentiate to xylem or phloem in the absence of active TDIF-PXY/TDR complexes. Vascular development is stimulated by light. After gemination, light induces photoautotrophic growth and photosynthesis. Consequently, the differentiation of vascular cells must occur to favor the transport of water and minerals from the soil to the green tissues and newly developing organs. Plants perceive light through the action of photoreceptors, which modulate the activity of the transcription factors (TFs) that orchestrate development. Despite the association between light signaling and vascular development, molecular mechanisms linking the two are unknown. Our work shows that vascular differentiation is inhibited in the dark by a mechanism that depends on PIF TFs. Dark-mediated accumulation of PIFs is necessary for *CLE44* induction and thus maintenance of undifferentiated vasculature. In illuminated environments, PIF inactivation by photoreceptors causes a decrease in *CLE44* expression. This *CLE44* decline, in turn, leads to reduced PXY/TDR signaling, which induces the xylem differentiation required to fulfill the water demands associated with photoautotrophic development.

RESULTS AND DISCUSSION

Light induces xylem differentiation in seedlings

When a seed germinates in darkness (skotomorphogenesis), it adopts etiolated growth, characterized by rapid elongation of the hypocotyl, maintenance of the apical hook to protect the shoot meristem, and inhibition of cotyledon greening and growth (Figure 1A). Here, growth depends on the energy stored in the endosperm nutrient reserves (Kornberg and Beevers, 1957; Penfield et al., 2004). Light perception guides the transition to photoautotrophic development, which inhibits hypocotyl elongation and promotes cotyledon greening and expansion for energy production via photosynthesis. This process is known as deetiolation.

Water is essential for photosynthesis, and so it must be transported from the root to the newly developing organs via vascular tissues. We hypothesized that during deetiolation, vasculature development must be stimulated by a light-dependent molecular mechanism. We tested this by defining the impact of light on changes to vascular tissues of 5-day-old seedlings grown in both light and dark conditions (Figures 1A–1G).

The vasculature of a 5-day-old *Arabidopsis* hypocotyl demonstrates a diarchic organization with two poles of xylem cells and, perpendicular to these, two poles of phloem cells in both light- and dark-grown conditions. A group of procambium or provascular cells occupy the space between xylem and phloem within the vascular cylinder (Figure S1A). Each xylem pole contains one protoxylem cell (outer) and one metaxylem (inner) cell. Protoxylem cells differentiate earlier in development and are characterized by the presence of annular or helical secondary cell wall

This is an open access article under the CC BY license (http://creativecommons.org/licenses/by/4.0/).

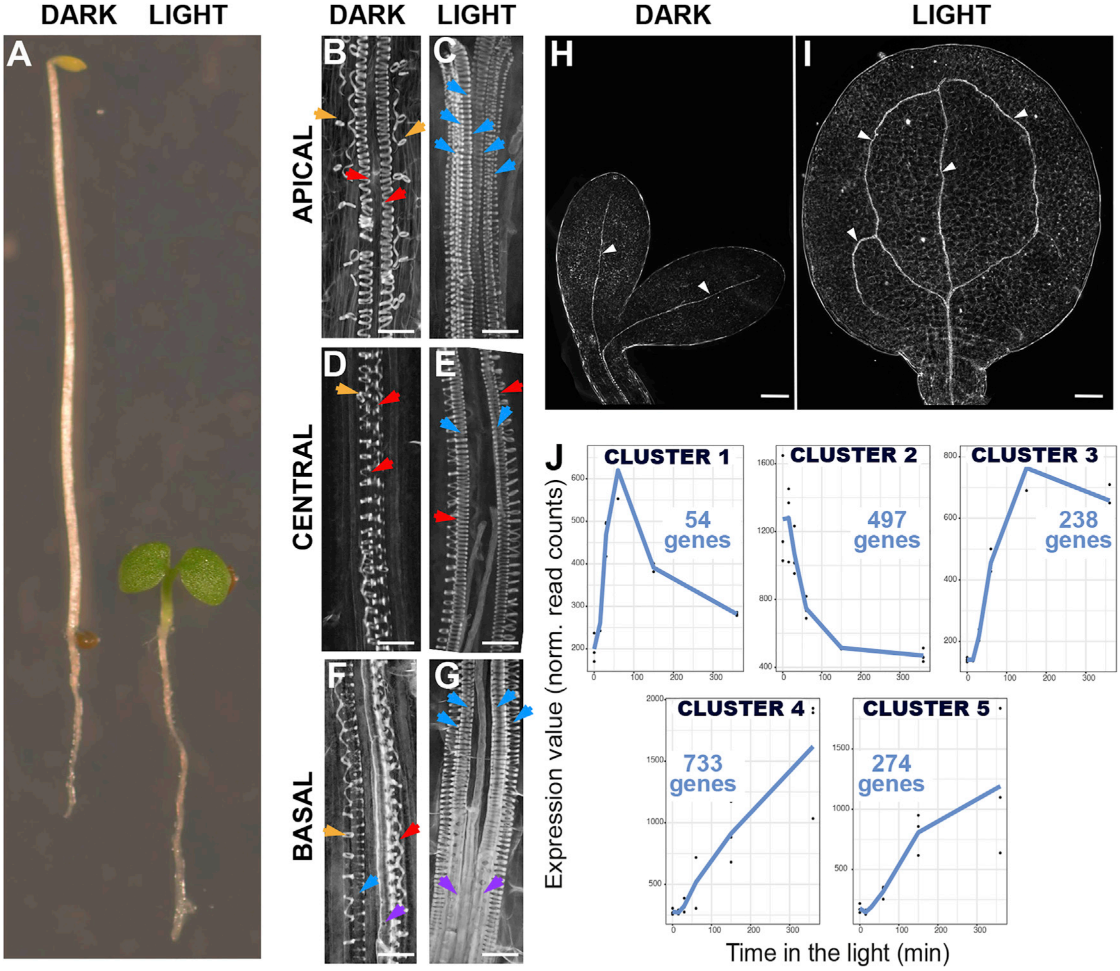

Figure 1. Light induces vasculature differentiation

(A) Five-day-old seedlings grown in the dark (left) and light (right).

(B–G) SCW deposition arrangements observed in hypocotyls of 5-day-old seedlings grown in the dark (B, D, and F) or light (C, E, and G). (B and C) Apical, (D and E) central, and (F and G) basal regions. Arrows highlight SCW arrangements; annular (orange), helical (red), reticulate (blue), pitted (purple). Scale bars: 10 μm.

(H and I) Differences in cotyledon vein differentiation (white arrowheads) between 5-day-old dark- (H) and light-grown (I) cotyledons. Scale bars: 100 μm.

(J) Genes clustered by transcriptional behavior during seedling deetiolation represented as average value. Data are representative of three independent experiments per time point. See also Data S1.

(SCW) patterning, while metaxylem cells differentiate later as cells with reticulate and pitted SCWs (Turner et al., 2007). Dark-grown seedling hypocotyls exhibited few mature reticulate and pitted metaxylem. If present at all, they were found only adjacent to the hypocotyl-root junction (Figure 1F). By contrast, in light-grown hypocotyls, reticulate metaxylem cells were present along the entire length of the hypocotyl apical-basal axis (Figures 1E and 1G). Thus, in dark-grown hypocotyls, metaxylem was discontinuous, whereas it was continuous in light-grown seedlings. This was most apparent in the middle region of the hypocotyl (Figure 1D). Moreover, the helices of SCW thickenings were looser in both proto and metaxylem in dark-grown seedlings than in those grown in the light (Figures 1B–1G). Importantly, the central procambium or provascular cells between xylem poles only differentiated to metaxylem in light-grown seedlings (Figure 1C). Xylem differentiation in cotyledons was also induced by light. Five-day-old seedlings exhibited cotyledons with a primary vein that connected with the hypocotyl vasculature and extended the length of the cotyledon proximodistal axis. Two secondary veins were also observed at this stage of development that branched from the primary, forming two continuous loops (Figure 1I). In dark-grown seedlings, continuous fully differentiated xylem files were observed only in the primary vein (Figure 1H). By contrast, light-grown seedlings also demonstrated fully differentiated xylem in the secondary loops (Figure 1I). Thus, light promotes xylem differentiation in the hypocotyl and cotyledon.

CLE44 expression is rapidly inhibited by light

To determine how vascular cell differentiation is induced by light, we performed a time course transcriptional analysis of 5-day-old seedlings grown in dark and following a 15-, 30-, 60-, 150-, or

CellPress
OPEN ACCESS

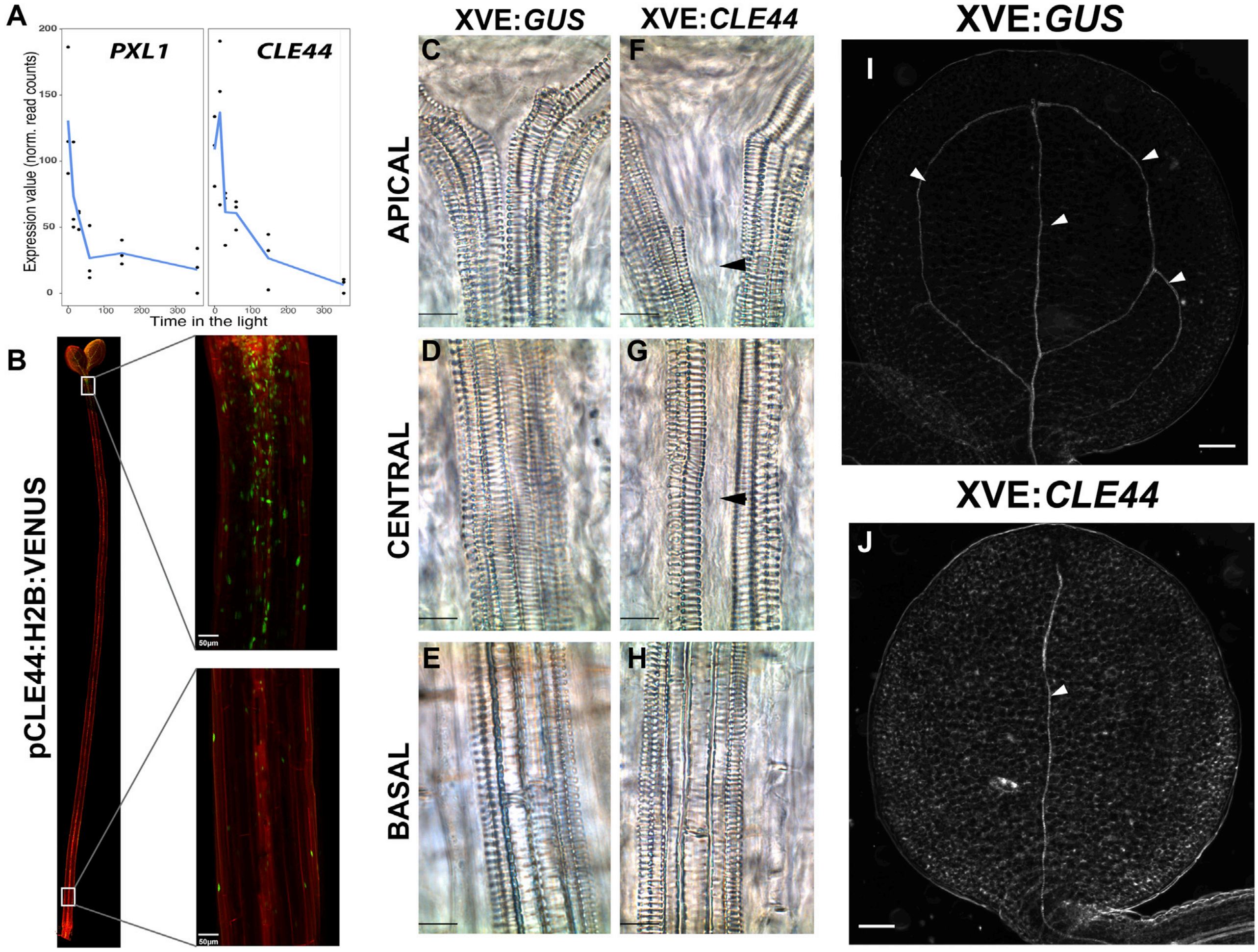

Figure 2. Light repression of CLE44 induces vascular differentiation

(A) Transcriptional behavior of *PXL1* and *CLE44* during deetiolation. Data are representative of three independent experiments per time point. See also Data S1.

(B) Confocal analysis of the p*CLE44*:H2B:VENUS transcriptional reporter in dark-grown seedlings. Image insets show the difference in *CLE44* expression between the apical and basal regions of the hypocotyl. Scale bars: 50 µm.

(C–H) Differences in xylem cell differentiation along the length of the hypocotyl between *GUS*- (C, D, and E) and *CLE44*- (F, G, and H) overexpressing seedlings grown in the light for 5 days. Scale bars: 10 µm.

(I and J) Differences in cotyledon vein differentiation (white arrowheads) between 5-day-old GUS (I) and *CLE44* (J) overexpressing seedlings grown in the light for 5 days. Scale bars: 100µm.

360-min exposure to white light (WL) (Figure S1B). Transcriptional changes were assessed via a two-regression-step approach (Conesa et al., 2006). To improve the identification of biologically meaningful expression trends, we used regression modeling to cluster genes with similar expression profiles (Conesa et al., 2006).

Five clusters were identified, one containing genes rapidly repressed by light, and four others showing different light-induction dynamics (Figure 1J; Data S1). Gene Ontology analysis suggested that clusters 2 and 4 exhibited clear functional specialization, while clusters 1 and 5 shared more ontologies with the others (Figure S1C; Data S2). Strikingly, we found that signaling components of the TDIF-PXY/TDR vascular-differentiation machinery were synchronously downregulated by light. Specifically, the receptor *PXL1* and its ligand *CLE44* were found among 497 early downregulated genes in cluster 2 (Figures 1J and 2A).

TDIF ligand is derived from CLE41 and CLE44 and perceived by PXY/TDR (Hirakawa et al., 2008), PXL1, and PXL2 receptors (Zhang et al., 2016). As TDIF triggers signaling, we focused our research on understanding its function in response to light. Unlike *CLE41*, *CLE44* expression was light responsive, as confirmed by qRT-PCR (Figure S1D). To examine *CLE44* spatial distribution along the hypocotyl, a 2,406-bp *CLE44* promoter region was fused to a bright nuclear-localized H2B:VENUS yellow fluorescent protein. Etiolated seedlings demonstrated an apical-basal distribution gradient of *CLE44* expression that peaked in the apical part of the organ (Figure 2B). As expected, *CLE44* showed an expression maximum in cells surrounding the vascular tissue.

CLE44 overexpression inhibits light-induced xylem differentiation

During secondary growth, one function of TDIF signaling is to repress xylem differentiation (Hirakawa et al., 2008; Ito et al., 2006). If the repression of *CLE44* by light is a requirement for light-induced xylem differentiation, maintaining high *CLE44* expression in the light may prevent xylem formation. To test this, an estradiol-inducible *CLE44* line was created (*XVE*:*CLE44*) (Zuo et al., 2000) (Figure S1E). Xylem differentiation was analyzed in *XVE*:*CLE44* and *XVE*:*GUS* (control) seedlings grown under constant light in the presence of 17β-estradiol. In controls, the hypocotyl demonstrated clear metaxylem differentiation in the central cells located between xylem poles, with a characteristic high degree of reticulated SCW deposition (Figures 2C–2E). Likewise, the cotyledons showed clear differentiation in the primary vein and distal secondary vein loops (Figure 2I), which were indistinguishable from those of untreated wild-type (WT) seedlings (Figure 1I). Thus, 17β-estradiol treatment alone did not affect vasculature differentiation. By contrast, seedlings overexpressing *CLE44* maintained the central provascular cells in an undifferentiated state along most of the hypocotyl apical-basal axis (Figures 2F–2H). The basal region alone demonstrated differentiation of the central cells (Figure 2H). Furthermore, xylem cells failed to differentiate in the cotyledon secondary veins that form the distal loops (Figure 2J). Repression of *CLE44* transcription by light is thus required for the induction of xylem differentiation occurring during photomorphogenesis.

CLE44 expression in the dark is regulated by PIFs

Light spectrum influences plant responses (Fankhauser and Chory, 1997); therefore, we considered that *CLE44* transcriptional regulation might depend on the perception of specific light wavelengths. Thus, we studied the effect of monochromatic lights on *CLE44* expression by exposing dark-grown seedlings to 6 h of blue, red, and far-red lights (BLs, RLs, and FRLs, respectively). Only BL induced reduction of *CLE44* expression to levels comparable with WL (Figures 3A and S1D). To better understand the role of light quality in the control of xylem differentiation, we measured the number of differentiated xylem cells present in the apical part of the hypocotyl and their degree of SCW deposition (annular, helical, or reticulate) in seedlings grown under each wavelength. We focused our analysis on the apical part for consistency and because it is the region of highest *CLE44* expression (Figure 2B). Monochromatic light treatments produced a reduction in the number of cells undergoing xylem differentiation in all cases compared with WL. However, seedlings grown in BL demonstrated more xylem cells and a higher degree of SCW deposition than the ones grown under RL and FRL (Figures 3B and S2A).

We reasoned that *CLE44* repression by light might be a direct consequence of photoreceptor activity. Thus, we studied *CLE44* expression levels in loss of function lines for CRY1 and CRY2 (blue), PHYB (red), and PHYA (far-red) photoreceptors grown in WL. *phyA* and *cry1* mutants demonstrated significantly elevated *CLE44* expression compared with WT, *phyB*, and *cry2* (Figure 3C). We also investigated the influence of *cry1*, *cry2*, *phyA*, and *phyB* mutations in hypocotyl xylem cell differentiation. All photoreceptor mutants had fewer differentiated xylem cells compared with WT. *cry1* mutants also displayed significantly lower degrees of SCW deposition (Figures 3D and S2B). Together, these data indicate that the light regulation of *CLE44* expression is principally controlled by BL via CRY1 signaling and correlates with xylem differentiation.

Photoreceptor signaling relies on the modulation of TF activity that controls the expression of light-responsive genes (Buti et al., 2020). We sought to identify TFs whose activity could explain *CLE44* light-transcriptional behavior. We considered that the synchronous behavior of the genes in each transcriptional cluster (Figure 1J) could be explained by the existence of common transcriptional regulators. *Ab initio* TF binding predictions were undertaken using the TF2Network tool, which predicts potential regulators for a set of co-expressed genes (Kulkarni et al., 2018). The algorithm identified putative regulators for genes in all clusters (Data S3). Cluster 2, which included *CLE44*, was predicted to be regulated by 65 TFs, with PIF3, PIF5, and PIF4 among the most significant ones, targeting 44, 62, and 153 genes, respectively (Figure 3E; Data S3). Light signaling converges in PIF TFs, which, in turn, inhibit photomorphogenic growth (Lucas and Prat, 2014). In the dark, PIFs accumulate to promote the expression of genes involved in skotomorphogenic growth. The perception of light leads to their inactivation (Park et al., 2004). Downregulation of PIFs upon light perception is key to proper transition to photomorphogenic development (Lucas et al., 2008). Hence, we hypothesized that PIF activity was necessary for the expression of *CLE44* in the dark. If correct, *CLE44* expression would occur in regions where *PIFs* are also expressed. We tested this by investigating the expression pattern of transcriptional reporters for *PIF3*, *PIF4*, and *PIF5*, which have contrasting functions in skotomorphogenesis (Lucas and Prat, 2014). All three demonstrated an apical-basal expression gradient with higher expression toward the cotyledons (Figure 3F). *PIF3*, *PIF4*, and *PIF5* expression thus overlapped with that of *CLE44* (Figure 2B). Moreover, we searched for the presence of PIF-binding elements in the *CLE44* promoter and identified seven E-BOX elements (−2,500 bp), of which two were PIF BINDING E-BOX elements (PBE-CATGTG) (Martínez et al., 2018; Zhang et al., 2013), implicating PIFs in regulating *CLE44* expression in the dark (Figure 3G). We confirmed that PIF4 binds to the first PBE-BOX in the *CLE44* promoter *in vitro* (Figure 3H). Next, we analyzed previously published chromatin immunoprecipitation sequencing (ChIP-seq) data for PIF4:FLAG-overexpressing plants (Pedmale et al., 2015), which showed high-confidence PIF4 binding enrichment over the *CLE44* promoter (Figure 3G). The intensity of the binding correlated with the location of the E-BOX elements in the *CLE44* promoter. To further validate the PIF4 binding to *CLE44* promoter, we performed ChIP-qPCR on light-grown seedlings overexpressing PIF4:FLAG. Four independent regions of the *CLE44* promoter consistently displayed enrichment in the antibody sample versus the control, confirming the ChIP-seq results from Pedmale et al. (2015) (Figure 3I). Finally, to determine the role of PIF on *CLE44* transcription, we analyzed *CLE44* transcript levels in dark-grown seedlings for single (*pif1*, *pif3*, *pif4*, and *pif5*), double (*pif45*), triple (*pif345*, *pif145*, *pif135*, and *pif134*), and quadruple PIF mutants (*pifq*; *pif3*, *pif4*, *pif5*, and *pif7*), as it has been shown that the

CellPress
OPEN ACCESS

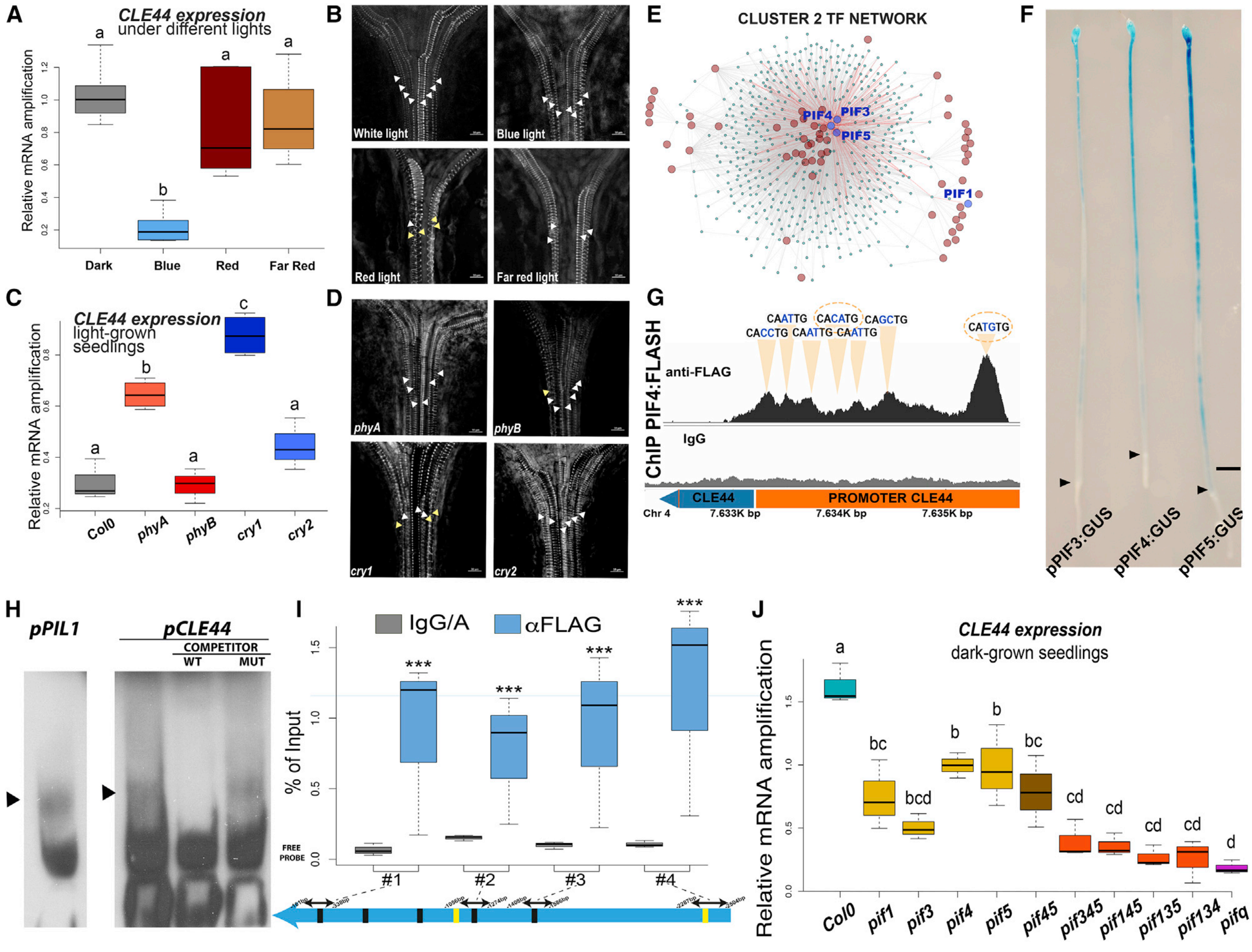

Figure 3. CLE44 expression is regulated by PIFs

(A) *CLE44* expression levels after 6 h of BL, RL, and FRL exposure. Data are representative of three independent experiments and three technical replicates per pair of primers. Values represent mean of expression ± SD. Letters indicate ANOVA + Tukey's honest significant difference (HSD) pairwise comparison test ($p < 0.05$).

(B) Orthogonal projections of confocal z stacks representative of hypocotyl xylem cell differentiation of seedling grown under WL (top left), BL (top right), RL (bottom left), and FRL (bottom right). Arrows highlight SCW arrangements; helical (yellow), reticulate (white). Scale bars: 10 μm.

(C) *CLE44* expression levels in photoreceptor mutants grown under continuous WL. Data are representative of three independent experiments and three technical replicates per pair of primers. Values represent mean of expression ± SD. Letters indicate ANOVA + Tukey's HSD pairwise comparison test ($p < 0.05$).

(D) Orthogonal projections of confocal z stacks representative of hypocotyl xylem cell differentiation in *phyA* (top left), *phyB* (top right), *cry1* (bottom left), and *cry2* (bottom right) mutants grown under continuous WL. Arrows highlight SCW arrangements; helical (yellow), reticulate (white). Scale bars: 10 μm.

(E) Hierarchical representation of the TF network for the genes in cluster 2. Labeled nodes represent the significant TFs identified by the TF2Network software. PIF TFs are indicated.

(F) Expression analysis of *PIF3*, *PIF4*, and *PIF5* transcriptional GUS reporter lines.

(G) PIF4-FLAG binding regions over CLE44 promoter identified via ChIP-seq. Immunoglobulin G (IgG) indicates the negative control. Orange triangles highlight the location of EBOX elements, dashed ellipses indicate PBE-BOX elements.

(H) Electrophoretic mobility shift assay (EMSA) showing interaction between PIF4 and the G-box elements of p*PIL1* (used as control), and the first PBE-BOX found on the *CLE44* promoter. WT = unlabelled WT probe, MUT = unlabelled mutated probe.

(I) Direct binding of PIF4 to the promoter of *CLE44* by ChIP-qPCR assays. Values obtained from three independent biological replicates and three technical replicates per pair of primers, were normalized to the input and compared against the IgG/A sample. Values represented as percentage of input ± SD. Comparisons between IgG/A and α-FLAG samples were made using Student's t test (*$p < 0.05$, **$p < 0.01$, ***$p < 0.001$, ns, not significant).

(J) *CLE44* expression levels in PIF mutants. Blue, WT; yellow, single; brown, double; orange, triple; purple, quadruple mutants grown in the dark. Data are representative of three independent experiments and three technical replicates per pair of primers. Values represent mean of expression ± SD. Letters indicate ANOVA + Tukey's HSD pairwise comparison test ($p < 0.05$).

PIF family shows functional redundancy (Leivar et al., 2008; Lorrain et al., 2009; Nozue et al., 2007; Shin et al., 2009). *CLE44* transcription was significantly reduced in all single and higher-order *PIF* mutants (Figure 3J). Thus, our data indicate that *CLE44* dark-dependent expression requires the combinatorial activity of different PIFs. Although, *CLE41* expression was not affected in *pifq* mutants grown in the dark compared with WT (Figure S2C), the fact that *CLE44* levels are ~10× higher than *CLE41* in etiolated seedlings indicates that CLE44 is the main TDIF precursor and is regulated in a PIF-dependent manner.

CLE44 induction in light-grown seedlings prevents xylem differentiation in WT and *pifq* mutants

As *PIF* genes are necessary for *CLE44* expression in the dark, we reasoned whether their genetic manipulation would affect xylem differentiation. Indeed, *pifq* mutants did have greater xylem differentiation levels than WT when grown in the dark, as fewer cells with annular deposition patterns were observed while more with helical and reticular wall arrangements were present (Figures 4A, 4C, and 4P). Regarding cotyledon venation, more than 80% of dark-grown WT seedlings demonstrated undifferentiated secondary veins. By contrast, only 5% remained undifferentiated in *pifq* cotyledons (Figures 4K, 4N, and 4Q). The phenotype of *pifq* mutants indicates that the repression of xylem differentiation in darkness is mediated by PIFs. In the light, all WT and *pifq* xylem files showed reticulate SCW patterns (Figures 4F, 4H, and S2D) and fully differentiated secondary cotyledon veins (Figure S2F).

To determine if the inhibition of xylem differentiation in the dark depends on the PIF-dependent expression of *CLE44*, we introgressed the *XVE*:*CLE44* transgene into the *pifq* mutant via crossing. The effect of *CLE44* induction in WT and *pifq* backgrounds grown in either dark or light was determined. *CLE44* induction in dark-grown WT seedlings led to fewer xylem files with reduced SCW complexity, as determined by more cells with annular depositions (Figures 4B and 4P). This suggests that the high *CLE44* expression observed in dark-grown WT seedlings does not fully saturate the PXY/TDR receptor. Dark-grown *pifq* seedlings complemented with *CLE44* via estradiol induction also showed reduced numbers of xylem-cell files with simpler cell walls (Figures 4D and 4P). In the cotyledons, *CLE44* induction inhibited the differentiation of the secondary veins observed in the *pifq* control sample (Figures 4N and 4O). These results are consistent with *CLE44* acting downstream of PIFs, as *CLE44* induction suppressed dark-grown *pifq* vascular phenotypes.

Induction of *CLE44* in both WT and *pifq* seedlings growing in the light also inhibited xylem differentiation in the central vascular cells in the apical hypocotyl (Figures 4G–4I, S2D, and S2E) and in secondary cotyledon veins (Figure S2F). Furthermore, *PIF4* overexpression was sufficient to inhibit regular xylem differentiation in the apical hypocotyl (Figure 4J), supporting the concept of *CLE44* being a PIF transcriptional target. Dark-grown *tdif-F* seedlings, which lack the activity of *CLE41*, *-42*, *-43*, and *-44* (Smit et al., 2020), show a higher degree of hypocotyl and cotyledon xylem differentiation than WT, highlighting the role of TDIF in this process (Figures 4E, 4M, and S2E).

In the 1960's, the botanist Katherine Esau described, in her book *Anatomy of Seed Plants*, how the extent of xylem differentiation depends on the context of organ growth (Esau, 1960). Specifically, xylem displays annular or low degrees of helical thickening in elongating organs, while mature organs show a higher degree of thickening that hinders cell elongation. The differences between dark- and light-grown seedlings described here provide one mechanism by which xylem differentiation is regulated. Given that the induction of *CLE44* expression inhibits xylem differentiation, we sought *prima facie* evidence that xylem differentiation itself is sufficient to impede the elongation process of a whole organ. Overexpression of *VASCULAR RELATED NAC-DOMAIN PROTEIN 7 (VND7)* induces transdifferentiation of various non-vascular cells into xylem vessels with SCW thickenings (Yamaguchi et al., 2010). To determine whether increased SCW deposition inhibited organ elongation, we induced *VND7* expression in dark-grown seedlings. Many hypocotyl cells underwent transdifferentiation to xylem vessels with reticulate SCW deposition (Figure S2G). Surprisingly, significant differences in hypocotyl elongation were not observed. Moreover, the induction of *CLE44* also did not induce hypocotyl elongation in WT and *pifq* plants (Figure 4R). These results support recent findings indicating that the epidermis, and not the vascular tissue, coordinates hypocotyl elongation (Kim et al., 2020; Robinson and Kuhlemeier, 2018).

Collectively, our data propose a model whereby the accumulation of PIFs in the dark inhibits vascular differentiation and SCW deposition by directly activating the expression of *CLE44*. CLE44 activates PXY/TDR family receptor kinases promoting maintenance of cambium identity and preventing precocious xylem differentiation. The presence of light rapidly promotes PIF inactivation, which, in turn, lowers *CLE44* transcription. Under such circumstances, xylem differentiation is induced, thus supporting water transport. Our data suggest that plants rely on *CLE44* modulation to prevent precocious xylem differentiation under sub-optimal photosynthetic conditions, and thus avoid the cell-wall deposition of carbon-rich polysaccharides.

Limitations of the study

The active form of CLE peptides is the result of proteolytic processing of precursor proteins (Ito et al., 2006). We show that *CLE44* transcription is induced in the dark in a PIF-dependent manner. However, we do not evaluate the effect of light in the proteolytic processing of TDIF precursors. While our analysis manipulating *CLE44* expression suggests that *CLE44* proteolytic processing does not represent a limiting step, it would be interesting to further investigate if this level of control is also subject to environmental regulation.

STAR★METHODS

Detailed methods are provided in the online version of this paper and include the following:

- KEY RESOURCES TABLE
- RESOURCE AVAILABILITY
  - Lead contact
  - Materials availability
  - Data and code availability

CellPress
OPEN ACCESS

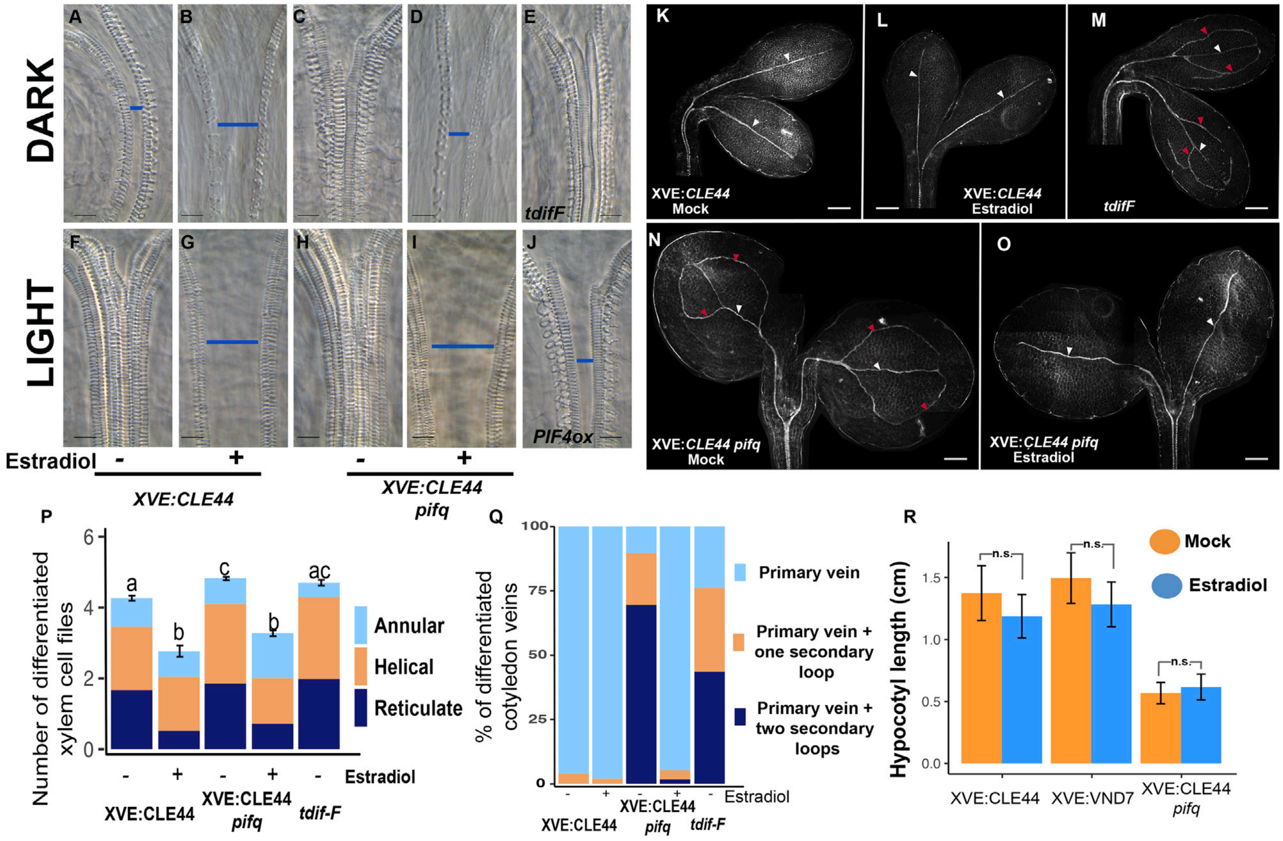

Figure 4. *CLE44* overexpression prevents xylem differentiation in WT and *pifq* mutants

(A–J) Differences in xylem cell differentiation in 5-day-old dark- (A–E) and light-grown (F–J) hypocotyls where *CLE44* and *PIF* expression is perturbed. Blue bars indicate undifferentiated provascular cells. Scale bars: 10 μm.

(A and B) XVE:*CLE44* grown in the dark. (A) Mock and (B) estradiol treated.

(C and D) XVE:*CLE44* in *pifq* background grown in the dark. (C) Mock and (D) estradiol treated.

(E) *tdifF* mutant grown in the dark.

(F and G) XVE:*CLE44* grown in the light. (A) Mock and (B) estradiol treated.

(H and I) XVE:*CLE44* in *pifq* background in the light.

(J) 35S:*PIF4*:FLAG grown in the light.

(K–O) Differences in cotyledon vein differentiation (white arrowheads) of dark-grown seedlings where *CLE44* and *PIF* expression is perturbed. White arrowheads, differentiated primary vein; red, differentiated secondary vein. Scale bars: 100 μm.

(K and L) XVE:*CLE44*. (K) Mock and (L) 17β-estradiol treated.

(M) *tdifF* mutant.

(N and O) XVE:*CLE44* in *pifq* background. (N) Mock and (O) 17β-estradiol treated.

(P) Differences in hypocotyl xylem differentiation between XVE:*CLE44*; XVE:*CLE44* in *pifq* and *tdifF* mutants grown in the dark and in the presence of mock and 17β-estradiol. Values ($n > 25$) represent mean of differentiated cells ± SE. Letters represent ANOVA + Tukey's HSD statistical test for total number of differentiated cells. Data S5 contains details of the sample size, mean, SE values, and ANOVA + Tukey's HSD comparisons.

(Q) Differences (as percentage) in cotyledon vein differentiation between XVE:*CLE44* and XVE:*CLE44* in *pifq* and *tdifF* mutants grown in the dark and in the presence of mock and 17β-estradiol ($n > 50$). Data S5 contains details of the sample size and percentages.

(R) Differences in hypocotyl length between XVE:*CLE44* and XVE:*CLE44* in *pifq* and XVE:*VND7* dark-grown seedlings in the presence of mock and 17β-estradiol. Values ($n > 20$) represent mean of hypocotyl length ± SD. Data S5 contains details of the sample size, mean, and SD values. Comparisons between mock and 17β-estradiol treated samples were made using Student's t test (*$p < 0.05$, **$p < 0.01$, ***$p < 0.001$, ns, not significant).

- EXPERIMENTAL MODEL AND SUBJECT DETAILS
  - *Arabidopsis* thaliana
  - Escherichia coli
- METHOD DETAILS
  - *Arabidopsis* growth conditions
  - Vector construction
  - RNA-seq sample collection and analysis
  - qRT-PCR analysis
  - Electrophoresis mobility shift assay (EMSA)
  - ChIP-qPCR analysis
  - GUS staining
  - Xylem differentiation analysis
  - Hypocotyl cross sections
  - Confocal analysis
  - Accession numbers
- QUANTIFICATION AND STATISTICAL ANALYSIS

SUPPLEMENTAL INFORMATION

Supplemental information can be found online at https://doi.org/10.1016/j.celrep.2022.111075.

ACKNOWLEDGMENTS

This work was supported by the start-up package and seedcorn funds from Durham University and by Gatsby Foundation research grants. S.G.'s research is funded via BBSRC Strategic LOLA (BB/V003534/1), and J.F.N.'s research is funded via DTP-BBSRC PhD studentship (BB/M011186/1). The authors thank Prof. Miguel Angel Blázquez (IBMCP-CSIC) for discussions and valuable suggestions that clearly improved the quality of the work and Dr. Eugenio G. Minguet for his advice setting the monochromatic light treatments.

AUTHOR CONTRIBUTIONS

S.G. conducted experiments and data analysis and provided comments. G.M.C.C. conducted data analysis and provided comments. J.F.N. conducted experiments and provided comments. J.P.E. provided comments and resources. M.d.L. conceived the project, conducted data analysis and experiments, and wrote the article. All authors read and approved the article.

DECLARATION OF INTERESTS

The authors declare no competing interests.

Received: July 12, 2021
Revised: January 15, 2022
Accepted: June 18, 2022
Published: July 19, 2022

REFERENCES

Ahmad, M., and Cashmore, A.R. (1993). HY4 gene of A. thaliana encodes a protein with characteristics of a blue-light photoreceptor. Nature *366*, 162–166. https://doi.org/10.1038/366162a0.

Bray, N.L., Pimentel, H., Melsted, P., and Pachter, L. (2016). Near-optimal probabilistic RNA-seq quantification. Nat. Biotechnol. *34*, 525–527. https://doi.org/10.1038/nbt.3519.

Buti, S., Hayes, S., and Pierik, R. (2020). The bHLH network underlying plant shade-avoidance. Physiol. Plant *169*, 312–324. https://doi.org/10.1111/ppl.13074.

Clough, S.J., and Bent, A.F. (1998). Floral dip: a simplified method forAgrobacterium-mediated transformation ofArabidopsis thaliana. Plant J. *16*, 735–743. https://doi.org/10.1046/j.1365-313x.1998.00343.x.

Conesa, A., Nueda, M.J., Ferrer, A., and Talón, M. (2006). maSigPro: a method to identify significantly differential expression profiles in time-course microarray experiments. Bioinformatics *22*, 1096–1102. https://doi.org/10.1093/bioinformatics/btl056.

Curtis, M.D., and Grossniklaus, U. (2003). A gateway cloning vector set for high-throughput functional analysis of genes in planta. Plant Physiol. *133*, 462–469. https://doi.org/10.1104/pp.103.027979.

Emami, S., Yee, M., and Dinneny, J.R. (2013). A robust family of Golden Gate Agrobacterium vectors for plant synthetic biology. Front. Plant Sci. *4*, 339. https://doi.org/10.3389/fpls.2013.00339.

Esau, K. (1960). Xylem: general structure and cell types. Anatomy of Seed Plants (New York: Wiley).

Etchells, J.P., Smit, M.E., Gaudinier, A., Williams, C.J., and Brady, S.M. (2016). A brief history of the TDIF-PXY signalling module: balancing meristem identity and differentiation during vascular development. New Phytol. *209*, 474–484. https://doi.org/10.1111/nph.13642.

Fankhauser, C., and Chory, J. (1997). Light control of plant development. Annu. Rev. Cell Dev. Biol. *13*, 203–229. https://doi.org/10.1146/annurev.cellbio.13.1.203.

Fu, C., Donovan, W.P., Shikapwashya-Hasser, O., Ye, X., and Cole, R.H. (2014). Hot fusion: an efficient method to clone multiple DNA fragments as well as inverted repeats without ligase. PLoS One *9*, e115318. https://doi.org/10.1371/journal.pone.0115318.t003.

Guo, H., Duong, H., Ma, N., and Lin, C. (1999). The Arabidopsis blue light receptor cryptochrome 2 is a nuclear protein regulated by a blue light-dependent post-transcriptional mechanism. Plant J. *19*, 279–287. https://doi.org/10.1046/j.1365-313x.1999.00525.x.

Hirakawa, Y., Shinohara, H., Kondo, Y., Inoue, A., Nakanomyo, I., Ogawa, M., Sawa, S., Ohashi-Ito, K., Matsubayashi, Y., and Fukuda, H. (2008). Non-cell-autonomous control of vascular stem cell fate by a CLE peptide/receptor system. Proc. Natl. Acad. Sci. USA *105*, 15208–15213. https://doi.org/10.1073/pnas.0808444105.

Ito, Y., Nakanomyo, I., Motose, H., Iwamoto, K., Sawa, S., Dohmae, N., and Fukuda, H. (2006). Dodeca-CLE peptides as suppressors of plant stem cell differentiation. Science *313*, 842–845. https://doi.org/10.1126/science.1128436.

Kim, S., Hwang, G., Kim, S., Thi, T.N., Kim, H., Jeong, J., Kim, J., Kim, J., Choi, G., and Oh, E. (2020). The epidermis coordinates thermoresponsive growth through the phyB-PIF4-auxin pathway. Nat. Commun. *11*, 1053. https://doi.org/10.1038/s41467-020-14905-w.

Kornberg, H.L., and Beevers, H. (1957). A mechanism of conversion of fat to carbohydrate in Castor beans. Nature *180*, 35–36. https://doi.org/10.1038/180035a0.

Kulkarni, S.R., Vaneechoutte, D., Van de Velde, J., and Vandepoele, K. (2018). TF2Network: predicting transcription factor regulators and gene regulatory networks in Arabidopsis using publicly available binding site information. Nucleic Acids Res. *46*, e31. https://doi.org/10.1093/nar/gkx1279.

Kumar, R., Ichihashi, Y., Kimura, S., Chitwood, D.H., Headland, L.R., Peng, J., Maloof, J.N., and Sinha, N.R. (2012). A high-throughput method for Illumina RNA-Seq library preparation. Front. Plant Sci. *3*, 202. https://doi.org/10.3389/fpls.2012.00202/abstract.

Lee, J., He, K., Stolc, V., Lee, H., Figueroa, P., Gao, Y., Tongprasit, W., Zhao, H., Lee, I., and Deng, X.W. (2007). Analysis of transcription factor HY5 genomic binding sites revealed its hierarchical role in light regulation of development. The Plant Cell *19*, 731–749.

Leivar, P., Monte, E., Oka, Y., Liu, T., Carle, C., Castillon, A., Huq, E., and Quail, P.H. (2008). Multiple phytochrome-interacting bHLH transcription factors repress premature seedling photomorphogenesis in darkness. Curr. Biol. *18*, 1815–1823. https://doi.org/10.1016/j.cub.2008.10.058.

Lorrain, S., Trevisan, M., Pradervand, S., and Fankhauser, C. (2009). Phytochrome interacting factors 4 and 5 redundantly limit seedling de-etiolation in continuous far-red light. Plant J. *60*, 449–461. https://doi.org/10.1111/j.1365-313x.2009.03971.x.

Lucas, M. de, and Prat, S. (2014). PIFs get BRright: PHYTOCHROME INTERACTING FACTORs as integrators of light and hormonal signals. New Phytol. *202*, 1126–1141. https://doi.org/10.1111/nph.12725.

Lucas, M. de, Davière, J.M., Rodríguez-Falcón, M., Pontin, M., Iglesias-Pedraz, J.M., Lorrain, S., Fankhauser, C., Blázquez, M.A., Titarenko, E., and Prat, S. (2008). A molecular framework for light and gibberellin control of cell elongation. Nature *451*, 480–484. https://doi.org/10.1038/nature06520.

Martínez, C., Espinosa-Ruíz, A., de Lucas, M., Bernardo-García, S., García, S.B., Franco-Zorrilla, J.M., and Prat, S. (2018). PIF4-induced BR synthesis is critical to diurnal and thermomorphogenic growth. EMBO J. *37*, e99552. https://doi.org/10.15252/embj.201899552.

Nelson, J.D., Denisenko, O., Sova, P., and Bomsztyk, K. (2006). Fast chromatin immunoprecipitation assay. Nucleic acids research *34*. e2–e2.

Nozue, K., Covington, M.F., Duek, P.D., Lorrain, S., Fankhauser, C., Harmer, S.L., and Maloof, J.N. (2007). Rhythmic growth explained by coincidence between internal and external cues. Nature *448*, 358–361. https://doi.org/10.1038/nature05946.

Park, E., Kim, J., Lee, Y., Shin, J., Oh, E., Chung, W.-I., Liu, J.R., and Choi, G. (2004). Degradation of phytochrome interacting factor 3 in phytochrome-mediated light signaling. Plant Cell Physiol. *45*, 968–975. https://doi.org/10.1093/pcp/pch125.

Pedmale, U.V., Huang, S.-S.C., Zander, M., Cole, B.J., Hetzel, J., Ljung, K., Reis, P.A.B., Sridevi, P., Nito, K., Nery, J.R., et al. (2015). Cryptochromes interact directly with PIFs to control plant growth in limiting blue light. Cell *164*, 233–245. https://doi.org/10.1016/j.cell.2015.12.018.

Penfield, S., Rylott, E.L., Gilday, A.D., Graham, S., Larson, T.R., and Graham, I.A. (2004). Reserve mobilization in the Arabidopsis endosperm fuels hypocotyl elongation in the dark, is independent of Abscisic Acid, and requires PHOSPHOENOLPYRUVATE CARBOXYKINASE1. Plant Cell *16*, 2705–2718. https://doi.org/10.1105/tpc.104.024711.

Reed, J.W., Nagpal, P., Poole, D.S., Furuya, M., and Chory, J. (1993). Mutations in the gene for the red/far-red light receptor phytochrome B alter cell elongation and physiological responses throughout Arabidopsis development. Plant Cell *5*, 147–157. https://doi.org/10.1105/tpc.5.2.147.

Robinson, S., and Kuhlemeier, C. (2018). Global compression reorients cortical microtubules in Arabidopsis hypocotyl epidermis and promotes growth. Curr. Biol. *28*, 1794–1802.e2. https://doi.org/10.1016/j.cub.2018.04.028.

Shin, J., Kim, K., Kang, H., Zulfugarov, I.S., Bae, G., Lee, C.-H., Lee, D., and Choi, G. (2009). Phytochromes promote seedling light responses by inhibiting four negatively-acting phytochrome-interacting factors. Proc. Natl. Acad. Sci. USA *106*, 7660–7665. https://doi.org/10.1073/pnas.0812219106.

Siligato, R., Wang, X., Yadav, S.R., Lehesranta, S., Ma, G., Ursache, R., Sevilem, I., Zhang, J., Gorte, M., Prasad, K., et al. (2016). MultiSite gateway-compatible cell type-specific gene-inducible system for plants. Plant Physiol. *170*, 627–641. https://doi.org/10.1104/pp.15.01246.

Smit, M.E., McGregor, S.R., Sun, H., Gough, C., Bågman, A.M., Soyars, C.L., Kroon, J.T., Gaudinier, A., Williams, C.J., Yang, X., et al. (2020). A PXY-mediated transcriptional network integrates signaling mechanisms to control vascular development in Arabidopsis. Plant Cell *32*, 319–335. https://doi.org/10.1105/tpc.19.00562.

Soneson, C., Love, M.I., and Robinson, M.D. (2016). Differential analyses for RNA-seq: transcript-level estimates improve gene-level inferences. F1000Res. *4*, 1521. https://doi.org/10.12688/f1000research.7563.2.

Toal, T.W., Ron, M., Gibson, D., Kajala, K., Splitt, B., Johnson, L.S., Miller, N.D., Slovak, R., Gaudinier, A., Patel, R., et al. (2018). Regulation of root Angle and gravitropism. G3 *8*, 3841–3855. https://doi.org/10.1534/g3.118.200540.

Turner, S., Gallois, P., and Brown, D. (2007). Tracheary element differentiation. Annu. Rev. Plant Biol. *58*, 407–433. https://doi.org/10.1146/annurev.arplant.57.032905.105236.

Yamaguchi, M., Goué, N., Igarashi, H., and Plant, M.O. (2010). VASCULAR-RELATED NAC-DOMAIN6 and VASCULAR-RELATED NAC-DOMAIN7 effectively induce transdifferentiation into xylem vessel elements under control of an induction system. Plant Physiol. *153*, 906–914.

Zhang, Y., Mayba, O., Pfeiffer, A., Shi, H., Tepperman, J.M., Speed, T.P., and Quail, P.H. (2013). A quartet of PIF bHLH factors provides a transcriptionally centered signaling hub that regulates seedling morphogenesis through differential expression-patterning of shared target genes in Arabidopsis. PLoS Genet. *9*, e1003244. https://doi.org/10.1371/journal.pgen.1003244.

Zhang, H., Lin, X., Han, Z., Qu, L.-J., and Chai, J. (2016). Crystal structure of PXY-TDIF complex reveals a conserved recognition mechanism among CLE peptide-receptor pairs. Cell Res. *26*, 543–555. https://doi.org/10.1038/cr.2016.45.

Zhang, R., Calixto, C.P.G., Marquez, Y., Venhuizen, P., Tzioutziou, N.A., Guo, W., Spensley, M., Entizne, J.C., Lewandowska, D., ten Have, S., et al. (2017). A high quality Arabidopsis transcriptome for accurate transcript-level analysis of alternative splicing. Nucleic Acids Res. *45*, 5061–5073. https://doi.org/10.1093/nar/gkx267.

Zuo, J., Niu, Q.W., and Chua, N.H. (2000). Technical advance: an estrogen receptor-based transactivator XVE mediates highly inducible gene expression in transgenic plants. Plant J. *24*, 265–273. https://doi.org/10.1046/j.1365-313x.2000.00868.x.

STAR★METHODS

KEY RESOURCES TABLE

REAGENT or RESOURCE	SOURCE	IDENTIFIER
Antibodies		
anti-FLAG-M2	Sigma	F3165; AB_259529
Protein A/G Magnetic Beads	Pierce	88802
Bacterial and virus strains		
Escherichia coli: ecloni	Lucigen	60107
Agrobacteria: GV3101pSoup	N/A	N/A
Biological samples		
Arabidopsis thaliana – Columbia-0	N/A	N/A
A. thaliana: *phyA-211*	Eirini Kaiserli Reed et al., 1993	*phyA-211*
A. thaliana: *phyB-9*	Dr. Eirini Kaiserli Reed et al., 1993	*phyB-9*
A. thaliana: *cry1-hb4-b104*	Dr. Eirini Kaiserli Ahmad and Cashmore, 1993	*cry1-hb4-b104*
A. thaliana: *cry2-1*	Dr. Eirini Kaiserli Guo et al., 1999	*cry2-1*
A. thaliana: *tdif-F*	Dr. Peter Etchells Smit et al., 2020	*tdif-F*
A. thaliana: *pifq*	Dr. Jaime F. Martinez García Leivar et al., 2008	*pifq*
A. thaliana: *pif1-2*	Dr. Jaime F. Martinez García Leivar et al., 2008	*pif1-2*
A. thaliana: *pif3-3*	Dr. Jaime F. Martinez García Leivar et al., 2008	*pif3-3*
A. thaliana: *pif4-1*	This lab Leivar et al., 2008	*pif3-3*
A. thaliana: *pif5-3*	Dr. Jaime F. Martinez García Leivar et al., 2008	*pif5-3*
A. thaliana: *pif1-2pif3-3pif4-1*	Dr. Jaime F. Martinez García Leivar et al., 2008	*pif1-2pif3-3pif4-1*
A. thaliana: *pif1-2pif3-3pif5-3*	Dr. Jaime F. Martinez García Leivar et al., 2008	*pif1-2 pif3-3 pif5-3*
A. thaliana: *pif1-2 pif4 pif5-3*	Dr. Jaime F. Martinez García Leivar et al., 2008	*pif1-2 pif4 pif5-3*
A. thaliana: *pif3-3 pif4 pif5-3*	Dr. Jaime F. Martinez García Leivar et al., 2008	*pif3-3 pif4 pif5-3*
A. thaliana: *pif4 pif5-3*	Dr. Jaime F. Martinez García Leivar et al., 2008	*pif4 pif5-3*
A. thaliana: *pif1-1*, *pif3-3*, *pif4-2*, *pif5-3*	Dr. Jaime F. Martinez García Leivar et al., 2008	*pif1-1*, *pif3-3*, *pif4-2*, *pif5-3*
A. thaliana: *35S:PIF4:FLASH*	Dr. Ullas Pedmalle Pedmale et al., 2015	*35S:PIF4:FLASH*
A. thaliana: pPIF3:GUS	NASC	N69166
A. thaliana: pPIF4:GUS	NASC	N69169
A. thaliana: pPIF5:GUS	NASC	N69172A
A. thaliana: pCLE44:H2B:VENUS	This paper	pCLE44:H2B:VENUS
A. thaliana: XVE:CLE44	This paper	XVE:CLE44
A. thaliana XVE:CLE44 in *pifq*	This paper	XVE:CLE44 in *pifq*
Chemicals, peptides, and recombinant proteins		
MS salts	Duchefa	M0222
MES	Duchefa	M1503
17β-estradiol	Sigma	E8875
X-Gluc	Apollo Scientific	BIMB1021
Critical commercial assays		
TNT Quick	Promega	L1170
LightShift[TM] Chemiluminescent EMSA	Thermo Scientific	20148
Streptavidin Magnetic beads	NEB	S1420S
RevertAid RT	Thermo Scientific	10161310
HiFi Polymerase	PCR Bio	PB10.41
Spurr Low Viscosity Embedding kit	Sigma	EM0300

(*Continued on next page*)

Continued

REAGENT or RESOURCE	SOURCE	IDENTIFIER
Deposited data		
Time course RNAseq data of seedlings during deetiolation	This paper	GSE178268
ChIPseq data for 35S:PIF4:FLASH	Pedmale et al., 2015	GSE68193
Oligonucleotides		
See Data S4 for a complete list of all oligonucleotides used in this study	IDT	N/A
Recombinant DNA		
pMDC7	Curtis and Grossniklaus, 2003	pMDC7
pMCY2	Emami et al., 2013	pMCY2
p2R3a-3xVenusYFP-OcsT	Siligato et al., 2016	*p2R3a-3xVenusYFP-OcsT*
Software and algorithms		
ImageJ 1.44o	National Institute of Health	https://imagej.nih.gov/ij/
R	R Core Team (2021)	https://www.R-project.org/

RESOURCE AVAILABILITY

Lead contact

Further information and requests for resources and reagents should be directed to and will be fulfilled by the lead contact, Miguel de Lucas (Miguel.de-lucas@durham.ac.uk).

Materials availability

Plasmids generated in this study have been deposited to Addgene, and *Arabidopsis* lines generated in this study have been deposited to NASC.

Data and code availability

- RNAseq data have been deposited at GEO and are publicly available as of the date of publication. Accession numbers are listed in the key resources table. This paper analyses existing, publicly available data. These accession numbers for the datasets are listed in the key resources table.
- This paper does not report original code.
- Any additional information required to re-analyze the data reported in this paper is available from the lead contact upon request.

EXPERIMENTAL MODEL AND SUBJECT DETAILS

Arabidopsis thaliana

All *Arabidopsis* plants used for this study were in Columbia-0 background. Photoreceptor mutants were kindly provided by Dr. Eirini Kaiserly (University of Glasgow, UK) and generated as follows: *phyA-211* (Reed et al., 1993), *phyB-9* (Reed et al., 1993), *cry1-hb4-b104* (Ahmad and Cashmore, 1993) and *cry2-1* (Guo et al., 1999). Combinations of PIF mutants were kindly provided by Dr. Jaime F. Martinez García (IBMCP-CSIC, Valencia, Spain) (Leivar et al., 2008). Transgenic plants expressing *pCLE44*:*H2B*:*3xVENUS* and *XVE*:*CLE44* were generated via Agrobacterium transformation (Clough and Bent, 1998). *XVE*:*CLE44* in the *pifq* background was generated via crossing. *pPIF3*:*GUS* (N69166), *pPIF4*:*GUS* (N69169) *and pPIF5*:*GUS* (N69172) expressing lines were obtained from NASC and *PIF4*:*FLASH* over-expressing lines were kindly provided by Ullas Pedmale (CSHL-New York, USA) (Pedmale et al., 2015).

Escherichia coli

E.cloni competent bacteria (Lucigen) were used for routine molecular biology. All bacteria were grown in LB medium (Melford). The medium was also supplemented with 25μg.mL^{-1} kanamycin (Melford), 100 μg.mL^{-1} carbenicillin (Melford), 50μg.mL^{-1}spectinomycin (Melford) and/or 20μg.mL^{-1}streptomycin (Melford) as required to maintain the different plasmids.

METHOD DETAILS

Arabidopsis growth conditions

For seedling analysis, seeds were surfaced sterilized and plated on Murashige and Skoog (1% MS) medium without sucrose. Seeds were stratified in dark for 3d at 4°C, and then transferred and kept vertical into a Sanyo growth chamber with a PAR light intensity of 40μmol.m^{-2}.s^{-1} illuminated by a daylight-white fluorescence lamp (FL40SS ENW/37; Panasonic) in a 24h-light cycle and 21°C of temperature. For light treatments, an ELEXIA lamp (Heliospectra) with 430nm, 660nm and 730nm LED diodes was used to provide BL, RL and FRL treatments at intensities mimicking the ones detected by the fluorescence lamps at those wavelengths (430nm = 5μmol.m^{-2}.s^{-1}, 660nm = 5μmol.m^{-2}.s^{-1} and 730nm = 1μm.m^{-2}.s^{-1}). For dark-grown seedlings, plates were covered with 3 layers of aluminum foil upon 6h of germination induction with WL. For all the experiments where the induction of the gene was performed via XVE system, sterile seeds were germinated on 1% MS media supplemented with 10μM of 17β-estradiol (SIGMA) for 5 days. Selection of transgenic seedlings was performed in 1% MS medium supplemented with 30μg.mL^{-1} hygromycin or via mCherry seed fluorescence (Emami et al., 2013).

Vector construction

All primers used in this study are listed in Data S4. For the construction of *pCLE44*:*H2B*:*VENUS* we first amplified the 3xVenus sequence from *p2R3a-3xVenusYFP-OcsT* (Siligato et al., 2016) using the primers 3xVENUS_F and 3xVENUS_R and introduced via hot fusion reaction (Fu et al., 2014) into HindIII linear *pMCY2* (Emami et al., 2013) destination plasmid to create *pMCY2_3xVenus*. HISTONE 2B nuclear protein was amplified from *Arabidopsis* cDNA using the primers H2B_F and H2B_R and inserted in the HindIII site via Hot Fusion reaction. Lastly, *CLE44* promoter was amplified from genomic Col-0 DNA using the primers pCLE44_H2B_3xVenus_F and R and inserted into the SapI site via Hot Fusion to create the final pCLE44:H2B:3xVenus_pMCY2 plasmid. For the 17β-estradiol inducible *CLE44* transgene, we amplified *CLE44* coding sequence using the primers CLE44_pMDC7_F and R. The amplified PCR was inserted via hot fusion reaction into the *pMCD7* plasmid (Curtis and Grossniklaus, 2003) digested with AscI and PacI, creating the plasmid CLE44_pMDC7.

RNA-seq sample collection and analysis

Arabidopsis seedlings (Col-0) were grown at 21°C, in constant darkness, for 5 days following germination induction in 1% MS medium without sucrose. Tissue from dark-grown seedlings was collected in a dark room under green light. Remaining seedlings were then exposed to 40μmol.m^{-2}.s^{-1} WL for 15, 30, 60, 150, or 360min. Seedlings were collected at these time points and immediately frozen in liquid nitrogen. Three biological replicates were taken at each time point. mRNA extraction and cDNA synthesis were done as described by (Kumar et al., 2012). The resulting cDNA samples were used to create a library compatible with Illumina sequencing (Kumar et al., 2012). Single-read sequencing was conducted on an Illumina HiSeq 2500 at 50bp SR. Reads were quality filtered as described (Toal et al., 2018) and mapped to the AtRTD2-QUASI transcriptome (Zhang et al., 2017) using Kallisto (Bray et al., 2016; Zhang et al., 2017). Kallisto was run using the default k-mer size of 31 bp, and with the following additional parameters: -b 30 –single -l 187 -s 81. Output files were processed using tximport (Soneson et al., 2016) (type = "kallisto", countsFromAbundance = "lengthScaledTPM)". The gene-level count values generated by tximport were then used to identify differentially expressed genes. maSigPro (Conesa et al., 2006) was used to identify genes which changed dynamically over the entire time course, taking all time points into account. Gene-level count values were normalized in DESeq prior to their use in maSigPro. We observed a tendency of the maSigPro package to declare genes expressed at extremely low levels as changing significantly over time. To address this, genes for which 2 or more of the biological replicates at any given time point had "0" values were removed from our analysis. The following parameters were used for the maSigPro analysis: design matrix = 5 degrees of freedom; p.vector (Q = 0.01, MT.adjust = "BH", counts = TRUE, theta = 2.814765), T.fit (step.method = "backward", alfa = 0.01); sigs(rsq = 0.5, vars = "all").Genes which exhibited similar expression profiles over the time course were clustered together using the "see.genes()" function within maSigPro (cluster.method = "hclust", cluster.data = 1, k = 5). maSigPro automatically determined the optimal number of clusters to group genes into; for our data, this was 9 clusters. However, many of the clusters had extremely similar expression profiles; therefore, we grouped similar clusters together to create 5 final clusters.

qRT-PCR analysis

cDNA was generated as described above. qRT-PCR reactions were run in a RotorgeneQ thermocycler and differential expression was calculated by obtaining the mean CT amplification values from three independent experiments (biological replicates) and from the average of three technical replicates per biological replicate. In each case, amplification was calculated relative to a PP2A control (AT1G69960). All possible pairwise comparisons were tested using ANOVA test followed by TUKEY HSD test ($p < 0.05$). Primer sequences used for the detection of each transcript are listed in Data S4.

Electrophoresis mobility shift assay (EMSA)

EMSAs were performed according to (Martínez et al., 2018) with minor modifications. DNA fragments for p*PIL1* (used as control) and the first PBE-BOX element found on p*CLE44* were analyzed by using 5′ biotinylated oligonucleotides (Data S4). PIF4 protein was synthesized using transcription and translation system (TNT-Promega) according to manufacturer's protocol and using a PCR fragment

containing PIF4_CDS under the control of the T7 promoter. Detection of the biotinylated DNA was performed using LightShift^{TM} Chemiluminescent EMSA (Thermo Scientific).

ChIP-qPCR analysis

35S:PIF4:FLASH seedlings were grown under constant WL conditions for 5 days. Chromatin immunoprecipitation (ChIP) was performed as previously described (Lee et al., 2007) with the following modifications. The crude nuclear pellet of three independent replicates was resuspended in nuclear lysis buffer and sonicated in a Covaris M220 (Woburn, MA, USA) focused-ultrasonicator for 6 min at 6°C with a 5% duty factor. The soluble chromatin solution was incubated with 1 µg of anti-FLAG (Sigma) and IgG/A magnetic beads over-night. Chromatin-antibody complexes were captured with protein A/G magnetic beads (Thermo Fisher Scientific, Waltham, MA, USA). De-crosslinking reaction was performed with Chelex slurry (Bio-Rad, Watford, UK) (Nelson et al., 2006). For the identification of the PIF4 regulated regions, primer pairs were designed to amplify four different regions along the CLE44 promoter. We then performed a comparative analysis between IgG/A and PIF4:FLAG bound chromatin using RotorgeneQ thermocycler. Differential enrichment was estimated a percentage of input.

GUS staining

Plant tissue was fixed in 90% acetone for 30min and washed twice with water before GUS staining. Seedlings were submerged in the GUS staining solution (50mM phosphate buffer, 0.2% Triton TX-100, 1.5mM potassium ferrocyanide, 1.5mM potassium ferricyanide, and 2mM X-Gluc (5-bromo4-chloro-3-indolyl b- D -glucuronide cyclohexylammonium salt dissolved in DMSO; Gold Biotechnology G1281C1) vacuum infiltrated for 5min, and incubated at 37°C in the dark for 2h. Seedlings were washed with 70% ethanol over-night and re-hydrated with a series of diluted ethanol (50, 25, 10% and water). Seedlings were then mounted with Hoyer's solution on microscope slides. The activity of the GUS reporter gene was observed under a Zeiss Axioscope 2 fiuorescence microscope.

Xylem differentiation analysis

Seedlings were incubated with clearing solution (1% SDS, 200mM NaOH) for 30min and rinsed twice with distilled water. Then transferred to chloral hydrate solution (40g chloral hydrate, 10mL glycerol, 20mL H_2O) over-night and then mounted in Hoyer's solution and observed under a Zeiss Axioscope 2 fiuorescence microscope. The analysis of cotyledon vasculature was performed using a dark field filter.

Hypocotyl cross sections

Five-day-old light-grown hypocotyls incubated overnight at 4°C in fixation buffer (2.5% glutaraldehyde + 2% paraformaldehyde in 0.2M phosphate buffer, pH 7). Dehydration was performed by incubating the sample for 2h in serial dilutions of ethanol (20, 40, 60, 80, 90, and 95%). The sample was plastic embedded by performing the following steps: 12h incubation in 7:1 ethanol: Spurr's resin (SIGMA), 12h incubation in 3:1 ethanol:Spurr's resin, 12h incubation in 100% Spurr's resin, and 12h incubation in Spurr's resin. The resin was polymerized at 70°C for 12h. Blocks were trimmed, and 4µm cross sections were produced with a Finesse ME+ (Thermo scientific) microtome. Toluidine blue staining (0.1% of Toluidine blue in 0.1M phosphate buffer, pH 6.8) was performed before microscopy analysis.

Confocal analysis

Samples were imaged using a Zeiss 800 with Airscan (Department of Biosciences, Durham University).

Accession numbers

Next-generation DNA sequencing raw and processed data have been deposited into the Gene Expression Omnibus (GEO) with accession number GEO: GSE178268.

QUANTIFICATION AND STATISTICAL ANALYSIS

Data for quantification of the differences in xylem differentiation (hypocotyl and cotyledon) are represented as mean ± standard error (SE). Number of hypocotyls or cotyledons (n), mean, percentage and SE for each xylem cell differentiation analysis are summarized on Data S5. RNAseq and qRT-PCR analysis were performed in three biological replicates (100-300 seedlings/replicate). qRT-PCR data quantification is presented as mean of expression ± standard deviation (SD). Statistical analyses, indicated in figure legends, were performed using R software. Comparisons between two groups were made using Student's t test (*$p < 0.05$, **$p < 0.01$, ***$p < 0.001$, ns = not significant). Comparisons between multiple groups were made using ANOVA + Tukey HSD test ($p < 0.05$). ANOVA + Tukey HSD tables are summarized on Data S5.

Cell Reports, Volume *40*

Supplemental information

Light regulates xylem cell differentiation

via PIF in *Arabidopsis*

Shraboni Ghosh, Joseph F. Nelson, Geoffrey M.C. Cobb, J. Peter Etchells, and Miguel de Lucas

Light regulates xylem cell differentiation via PIF in Arabidopsis

Shraboni Ghosh[a], Joseph F. Nelson[a], Geoffrey Cobb[ab], J. Peter Etchells[a] and Miguel de Lucas[a]

SUPPLEMENTARY FIGURES

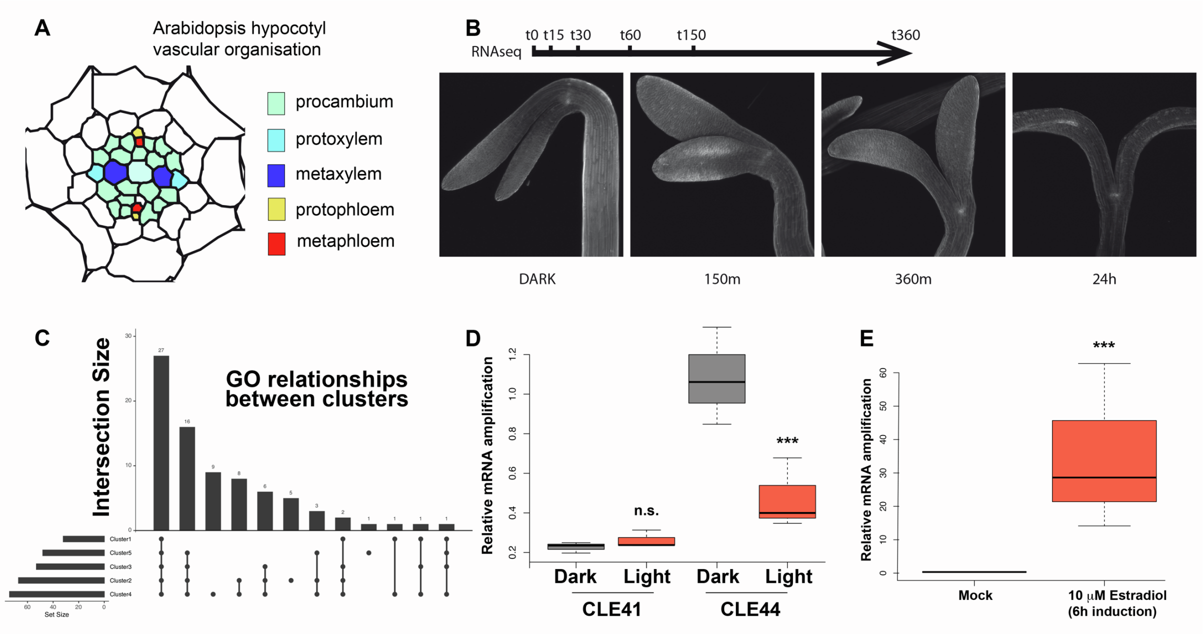

Figure S1. Related to Figure 1, 2 and 4

(A) Cartoon of a cross section of the Arabidopsis hypocotyl showing the different cell types forming the vascular tissue.

(B) Diagrammatic representation of the RNAseq experimental set up. Samples were taken at different time points over the course of 5-day old seedling deetiolation (Dark, 15, 30, 60, 150 and 360min, three replicates per time point). Related to Figure 1J and Data S1.

(C) Upset plot describing the relationships between enriched gene-ontology categories across the five different gene clusters. Related to Data S2.

(D) Comparison between *CLE41 and CLE44* expression levels in seedlings grown in the dark and after 6h of light. Data are representative of three independent experiments and three technical replicates per pair of primers. Values represent mean of expression ± SD. Letters indicate Student's t.test ($*p < 0.05$, $**p < 0.01$, $***p < 0.001$, ns = not significant). Related to Figure 1 and 2.

(E) Confirmation of *CLE44* induction in XVE:*CLE44* seedlings. Data are representative of three independent experiments and three technical replicates per pair of primer. Values represent mean of expression ± SD. Letters indicate Student's t.test ($*p < 0.05$, $**p < 0.01$, $***p < 0.001$, ns = not significant). Related to Figure 2 and 4.

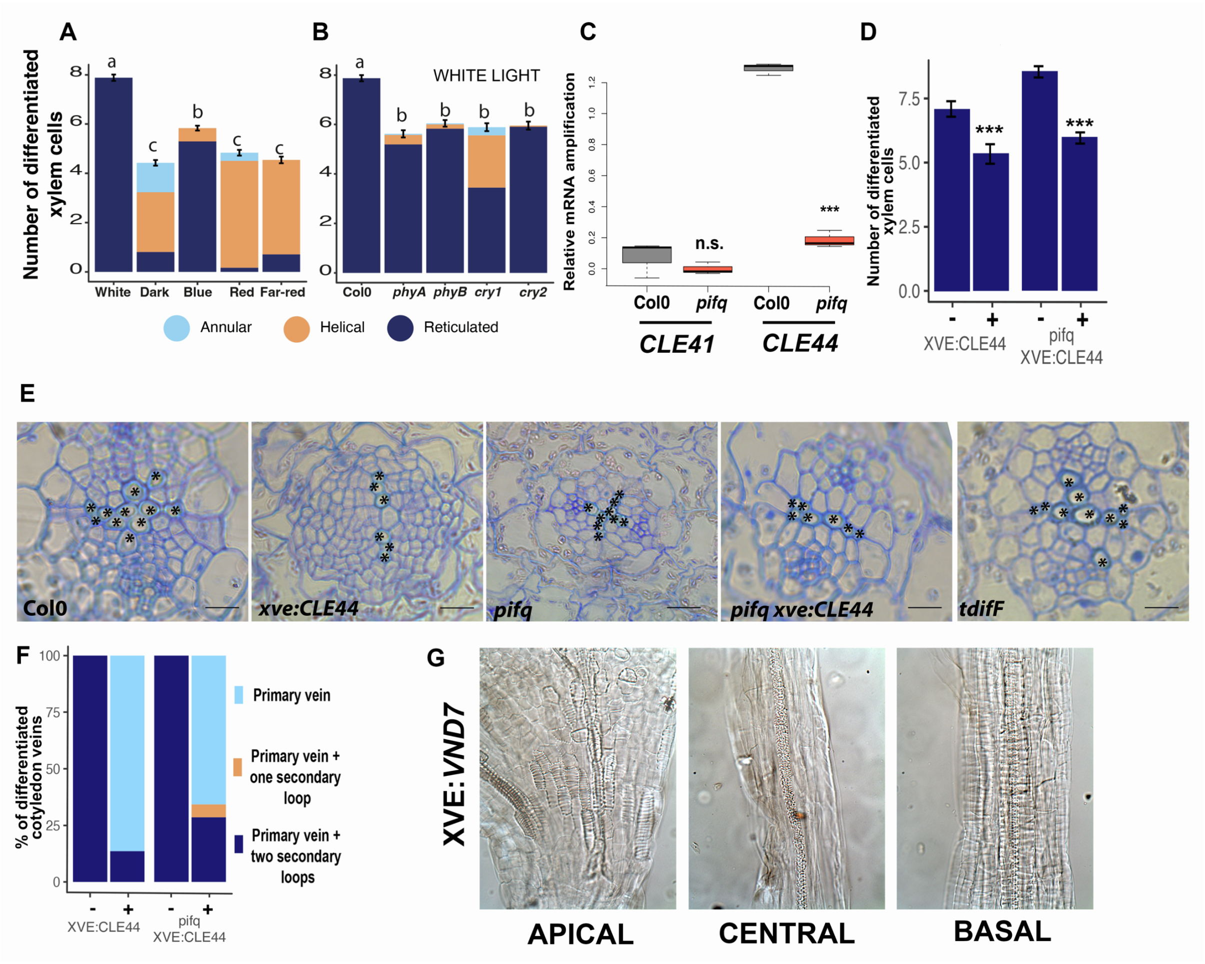

Figure S2. Related to Figure 4.

(A) Differences in hypocotyl xylem differentiation between seedlings treated with different wavelengths of monochromatic light. Values (n>24) represent mean of differentiated cells ± SE. Letters represent ANOVA + Tukey HSD statistical test. Data S5 contains details of the sample size, mean, SE values and ANOVA + Tukey HSD comparisons.

(B) Differences in hypocotyl xylem differentiation between photoreceptor mutants grown under white light. Values (n>18) represent mean of differentiated cells ± SE. Letters represent ANOVA + Tukey HSD statistical test. Data S5 contains details of the sample size, mean, SE values and ANOVA + Tukey HSD comparisons.

(C) Differences between *CLE41* and *CLE44* expression levels in Col0 (WT) and *pifq* mutant seedlings grown in the dark. Data are representative of three independent experiments and three technical replicates per pair of primer. Values represent mean of expression ± SD. Letters indicate Student's t.test (∗p < 0.05, ∗∗p < 0.01, ∗∗∗p < 0.001, ns = not significant).

(D) Differences in hypocotyl xylem differentiation between XVE:*CLE44* and XVE:*CLE44* in *pifq* seedlings grown in the light and in the presence of mock and 17ß-estradiol. Values (n>9) represent mean of differentiated

cells ± SE. Letters represent ANOVA + Tukey HSD statistical test. Data S5 contains details of the sample size, mean, SE values and ANOVA + Tukey HSD comparisons.

(E) Hypocotyl plastic cross sections showing the differences in xylem cell differentiation between Col0, XVE:*CLE44*, *pifq*, XVE:*CLE44 pifq* and *tdif-F* grown for 5-days in the presence of 17ß-estradiol. Xylem differentiated cells are indicated with and asterisk.

(F) Differences (as percentage) in cotyledon vein differentiation of photoreceptor mutants grown in the light. Data S5 contains details of the sample size and percentages

(G) DIC image showing the ectopic xylem differentiation caused by the induction of VND7 in different regions (apical, central and basal) of dark-grown hypocotyls.
